# “Modified sandwich” technique in the surgery of acute type A aortic dissection

**DOI:** 10.1186/s43044-025-00651-1

**Published:** 2025-06-10

**Authors:** Jiajie Kong, Tong Liu, Shuqiang Xi, Zhaobin Li, Zeyue Jin, Fan Yang, Zhe Zhu, Lei Liu

**Affiliations:** 1 Department of Cardiac Surgery, Hebei Medical University Third Hospital, Shijiazhuang, Hebei People’s Republic of China; 2 Department of Cardiac Surgery, Affiliated Hospital of Guangdong Medical University, Zhanjiang, Guangdong People’s Republic of China

**Keywords:** Acute type A aortic dissection, Artificial vascular slice, Modified sandwich method

## Abstract

**Background:**

Acute type A aortic dissection is a rapidly progressive and life-threatening condition. Without timely surgical intervention, the mortality rate can reach up to 50% within the first 48 h. Although surgery remains the primary effective treatment, it is associated with significant complexity and potential risks, particularly in managing the aortic root, where both intraoperative and postoperative bleeding complications are common. This study aims to evaluate the efficacy of the modified “sandwich” technique using a synthetic vascular patch for aortic root reconstruction in acute type A aortic dissection surgery.

**Methods:**

A retrospective analysis was conducted on the clinical data of 28 patients with acute type A aortic dissection who underwent aortic root reconstruction using the modified “sandwich” technique with synthetic vascular patches at the Department of Cardiovascular Surgery, the Third Hospital of Hebei Medical University, from October 2020 to November 2022. All patients underwent surgical treatment, during which the modified “sandwich” technique was applied for aortic root reconstruction. Statistical analysis was performed on operative time, cardiopulmonary bypass (CPB) time, aortic cross-clamp (ACC) time, postoperative drainage volume, perioperative mortality, and complications.

**Results:**

All 28 patients underwent successful surgery with a cardiopulmonary bypass (CPB) time of 265.0 (210.0–322.5) min, an aortic cross-clamping (ACC) time of 151.0 (112.0–209.0) min, and a drainage flow rate of 237.5 (126.0–297.0) mL at 12 h postoperatively. There were 2 (7.1%) perioperative deaths caused by renal failure, ischemia in 1 case, and coronary artery causes in 1 case. Postoperative complications included reopening of the chest for hemostasis in 1 case (3.6%) for reasons unrelated to the vascular anastomosis, hemodialysis in 3 cases (10.7%), paraplegia in 1 case (3.6%), and cerebral infarction resulting in impaired mobility of the left upper extremity in 1 case (3.6%). Tracheotomy was performed in 1 case (3.6%), and the duration of mechanical ventilation was 89 (48.0–165) h. Among the 26 recovered patients reviewed with aortic enhancement CT before discharge, the artificial vascular anastomosis had smooth blood flow, though 1 case still had residual entrapment in the sinus of the aorta.

**Conclusion:**

In acute type A aortic dissection surgery, the “modified sandwich” technique using an artificial vascular sheet for aortic root shaping is simple, effective, and easy to master. This method can reduce anastomotic blood seepage and prevent anastomotic tear and bleeding, making it worth recommending for clinical application.

## Background

Acute type A aortic dissection is a highly aggressive and life-threatening condition. According to Li et al. [1], if surgery is not performed promptly, patients face a mortality rate as high as 50% within 48 h. Nonetheless, the procedure itself is complex and high-risk, and the complete resection of the root lesion in the aortic wall, aimed at eliminating residual entrapment, is difficult to achieve in most cases. Therefore, reinforcing the lesion-affected and fragile proximal aortic wall to establish a strong anastomotic zone is crucial. This step is essential to prevent pinhole tearing of the anastomosis, rupture bleeding, or malignant coronary events caused by sustained pressure increases within the residual entrapment, ensuring procedural safety.

Since the 1970 s, a series of techniques for aortic root reinforcement and prevention of bleeding have been developed, including simple continuous suture apposition to fix separated inner and outer membranes, the sandwich technique and its various modifications, internalization of the outer membrane, flip anastomosis of artificial blood vessels, and the use of medical adhesives [2–5]. Prof. Liu Kexiang, a scholar from our country, used an artificial vascular sheet with a width of 1.5–2.0 cm for sandwich reinforcement and root molding, which effectively closed the proximal remnant of the sandwich and significantly reduced the risk of intraoperative bleeding and false lumen compression of the coronary artery [6]. However, the steps of continuous horizontal mattress transmural foldback suture at the lower edge of the sandwich and longitudinal suture between the upper and lower edges of the artificial vascular sheet are cumbersome and time-consuming. Thus, we simplified the suture fixation method of the artificial vascular sheet sandwich and still achieved good clinical efficacy. We now present our treatment experience and discuss the effectiveness of this method for clinical reference.

## Methods

### Surgical techniques

**Fig. 1:**
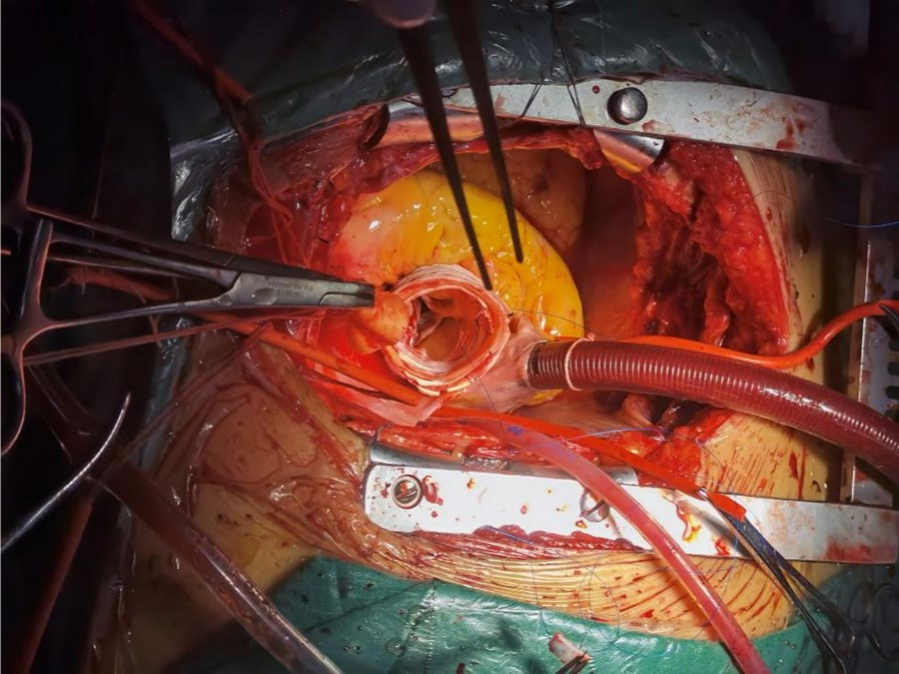
Completion of root treatment

A median chest incision was performed to longitudinally split the sternum and access the chest. Three cephalic and brachial arteries were then dissected and freed. Extracorporeal arterial perfusion was conducted using a single pump and two cannulas: one through the femoral artery for extracorporeal circulation and the other for selective cerebral perfusion. After establishing extracorporeal circulation, cooling was initiated. The ascending aorta was occluded and longitudinally dissected. Histidine-tryptophan-ketoglutarate (HTK) myocardial protection fluid was infused through the right and left coronary artery openings. The thrombus in the entrapment was removed, the ascending aorta was transected 1.5–2 cm above the sinus-tubular junction, and the aortic root was dissected to the right and left coronary arteries above the main trunks. Initially, the sinus was examined for entrapment, avulsion of the junction, and involvement of the coronary openings. The root was then injected with water to verify the integrity of the inner and outer membranes and to assess any lesions of the aortic valve. If the lesion was suitable for root reinforcement molding, the artificial blood vessel was selected based on the diameter of the root ascending aorta and sinotubular junction. Three 1.5-cm-wide vascular rings were cut from the artificial vessel. One ring was placed with the intima against the proximal dissected end of the ascending aorta, while the other two strips were cut longitudinally and attached to the outer side of the dissected vessel. The remaining artificial vessels were trimmed into appropriately shaped and sized spacers and placed between the inner and outer membranes of the avulsed coronary sinus and sinotubular junction. Each vascular piece was adjusted to fit properly against the aortic wall. The lower edge of the sandwich was then secured with a three-needle 4–0 Prolene suture in three equal interrupted mattress seams above the sinotubular junction (noting that the first stitch was placed over the opening of the left coronary artery). One of the sutures was then used to create a continuous horizontal mattress suture along the level of the sinotubular junction, followed by another continuous horizontal mattress suture along the upper edge of the sandwich. The medial prosthetic vascular sheet was examined and trimmed as necessary if it affected the coronary artery openings. In cases where the leaflet junction was affected by the sandwich and avulsion led to aortic closure insufficiency, a 4–0 double-needle mattress suture with a spacer was used to suspend the aortic valve junction. A water injection test was performed to observe aortic valve closure. If any part of the coronary opening was involved in the sandwich, it was fixed with interrupted mattress sutures using a 5–0 Prolene thread. The upper edge of the sandwich was then neatly trimmed with scissors, ensuring that the layers of the artificial vascular sheet and the wall of the autologous aorta were properly aligned. Care was taken not to damage the upper edge of the suture line (see Fig. 1). The distal end of the sandwich was treated with either classic full arch replacement with stenting and elephant trunk surgery, simple ascending artery replacement, or half-arch replacement, depending on the extent of sandwich involvement. Selective cerebral perfusion was performed during a deep hypothermic shutdown cycle. Following distal treatment, the proximal end of the four-branched artificial vessel was anastomosed to the proximal ascending aorta using 3–0 Prolene sutures. After venting, the blocking clamp was removed, extracorporeal circulation was gradually discontinued, and cardioversion was performed.

Compared with the conventional David procedure, the modified sandwich technique eliminates the need for complete resection of the diseased aortic sinuses and subsequent valve reimplantation into the prosthetic graft, resulting in significantly shorter aortic cross-clamp times (151.0 [112.0–209.0] min vs. 170 ± 65 min) [27]. Moreover, the modified sandwich technique involves only 4–0 pledgeted suspension of the detached commissures while preserving the native valve geometry. This approach simplifies the root reconstruction process and reduces operative duration.

### Statistical analysis

Statistical analysis was conducted using SPSS 22.0 software. Measurements with a normal distribution were expressed as mean ± standard deviation (mean ± SD). For data not following a normal distribution, values were presented as median and interquartile range (IQR). Categorical data were reported as frequency or percentage (%).

## Results

From October 2020 to November 2022, 28 patients with acute type A aortic dissection underwent surgical treatment in the Department of Cardiovascular Surgery at the Third Hospital of Hebei Medical University. Root shaping was performed using the “modified sandwich” method with artificial vascular sheets during the operation. Among the 28 patients, 18 were male and 10 were female. The average BMI was (27.29 ± 4.25) kg/m^2^. Twenty-two patients had hypertension, 1 had diabetes mellitus, and 1 had coronary artery disease. The left ventricular ejection fraction (EF) was 61.0 ± 4.8%. Preoperative routine examinations revealed: serum creatinine (Cr) 92.70 (57.58–109.72) μmol/L, urea 2.375 (5.05–7.425) mmol/L, total bilirubin 17.94 (13.02–30.96) μmol/L, alanine aminotransferase (ALT) 11.5 (15.0–26.5) U/L, and aspartate aminotransferase (AST) 34.5 (22.0–45.25) U/L. All patients underwent preoperative aortic CTA and echocardiography to establish the diagnosis, assess the extent of entrapment involvement, evaluate vascular involvement of each important branch, measure the diameters of each aortic segment, and select the site for arterial cannulation. The Ethics Committee of the Third Hospital of Hebei Medical University approved the study and granted an exemption from written informed consent.

**Fig. 2:**
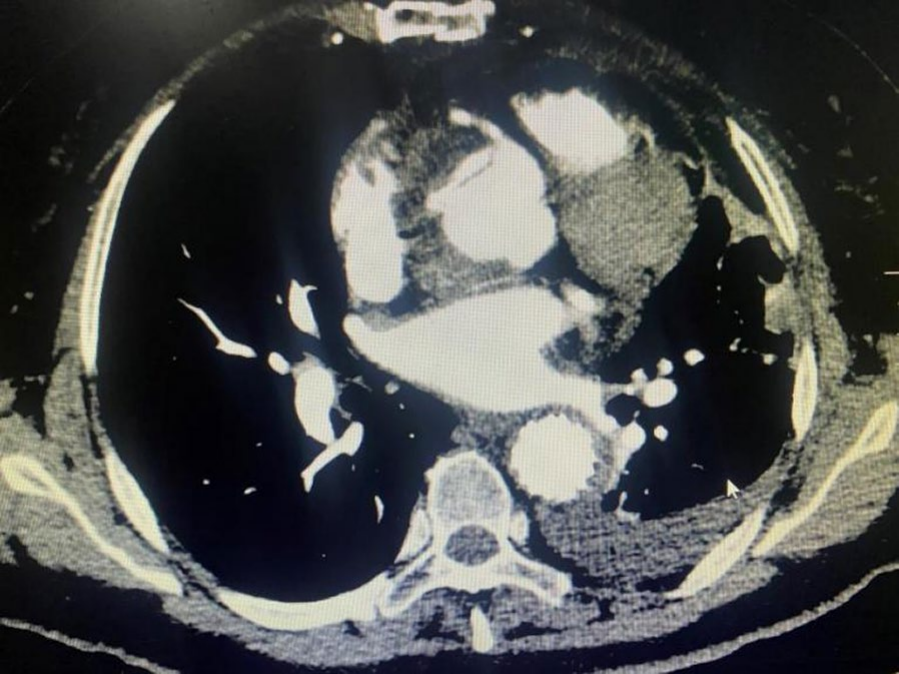
Residual entrapment in the sinus of the aorta

In our cohort, 28 cases of acute type A aortic dissection underwent intraoperative “modified sandwich” root shaping with artificial vascular slices. Among these cases, 24 patients received total arch replacement with stenting and elephant trunk surgery, 4 patients had ascending aorta replacement with a small curvature of the arch under deep hypothermic shutdown, and 1 patient required coronary bypass grafting due to severe involvement of the right coronary artery opening. All 28 patients successfully completed their surgical treatment. The operative time was 9.5 (8.0–12.2) hours, including selective cerebral perfusion time of 23.0 (8.0–35.0) minutes, intraoperative CPB time of 265.0 (210.0–322.5) minutes, and ACC time of 151.0 (112.0–209.0) minutes. Postoperative awakening time for 21 patients was 36.0 (33.0–81.5) hours, endotracheal intubation time was 88.6 (55.5–172.1) hours, and postoperative ICU stay was 124.7 (89.9–185.1) hours. There were 2 perioperative deaths (7.1%): one due to renal failure and abdominal ischemia, and the other due to coronary artery causes. At 24 h postoperatively, serum creatinine (Cr) was 104.19 (81.70–149.30) μmol/L, urea was 9.76 (6.44–13.45) mmol/L, total bilirubin was 52.28 ± 24.75 μmol/L, alanine aminotransferase (ALT) was 24.5 (19.0–43.0) U/L, and aspartate aminotransferase (AST) was 56.0 (38.0–114.0) U/L in 26 patients. Postoperative complications included reopening of the chest for hemostasis in 1 case (3.6%) due to reasons unrelated to the vascular anastomosis, hemodialysis in 3 cases (10.7%), paraplegia in 1 case (3.6%), cerebral infarction with impaired mobility of the left upper extremity in 1 case (3.6%), and tracheotomy in 1 case (3.6%) (see Table 1). Of the 26 recovered patients, aortic enhancement CT prior to discharge showed that blood flow through the prosthetic anastomosis was smooth, although 1 case still had residual entrapment in the aortic sinus (see Fig. 2).

**Table 1:** Surgical and postoperative outcomes of patients undergoing modified “sandwich” technique for aortic root reconstruction in acute type A aortic dissection

Variable	Value/Outcome
Surgical procedures	
Total arch replacement + FET	24 cases
Ascending aorta + hemiarch replacement	4 cases
Coronary artery bypass grafting	1 case
Operative time	9.5 (8.0–12.2) hours
Selective cerebral perfusion time	23.0 (8.0–35.0) minutes
Cardiopulmonary bypass time (CPB)	265.0 (210.0–322.5) minutes
Aortic cross-clamp time (ACC)	151.0 (112.0–209.0) minutes
Postoperative recovery	
Time to awakening	36.0 (33.0–81.5) hours
Duration of intubation	88.6 (55.5–172.1) hours
ICU stay	124.7 (89.9–185.1) hours
Perioperative mortality	2 cases (7.1%)
Causes of death	Renal failure with visceral ischemia (1 case) and coronary artery-related (1 case)
Postoperative laboratory results (24 h)	
Serum creatinine (Cr)	104.19 (81.70–149.30) μmol/L
Urea	9.76 (6.44–13.45) mmol/L
Total bilirubin	52.28 ± 24.75 μmol/L
ALT	24.5 (19.0–43.0) U/L
AST	56.0 (38.0–114.0) U/L
Postoperative complications	
Reexploration for bleeding	1 case (3.6%), unrelated to vascular anastomosis
Hemodialysis	3 cases (10.7%)
Paraplegia	1 case (3.6%)
Cerebral infarction with left arm impairment	1 case (3.6%)
Tracheostomy	1 case (3.6%)
Follow-up imaging (predischarge)	
Patent artificial vascular anastomosis	26 cases
Residual dissection in aortic sinus	1 case

## Discussion

Acute type A aortic dissection is an aggressive condition with a poor prognosis, challenging treatment, and a high propensity for serious complications, including poor organ perfusion and acute kidney injury [7–10]. With the rapid advancements in technology and material science, the surgical success rate for acute type A aortic dissection has improved significantly. According to the International Registry of Acute Aortic Clamping, the surgical success rate has reached 90%, while the mortality rate has decreased from 25% in 1995 to 18% in 2013[11]. However, a multicenter study revealed that Chinese patients with acute type A aortic dissection experience a longer interval between onset and hospital arrival but have relatively lower early mortality rates. This study suggests the presence of a significant survivor bias among Chinese patients with acute type A aortic dissection [12]. This implies that patients who reach the hospital alive for treatment may possess certain physiological or pathological advantages, while those who do not reach the hospital in a timely manner during the early stages may be excluded from the statistics, potentially leading to an underestimation of the overall mortality rate. The relatively low proportion of patients with acute type A aortic dissection undergoing surgical treatment highlights the urgent need to enhance the promotion and accessibility of surgical interventions. This underscores the importance of improving surgical adoption rates and expanding the availability of these life-saving procedures.[13].

One of the keys to achieving surgical success is the prevention of uncontrollable intraoperative hemorrhage, particularly in the proximal anastomosis at the aortic root, where the risk of hemorrhage is significantly increased by the presence of residual entrapment and a high-pressure environment. Management of the aortic root entails the following: effective closure of the false lumen, protection of the valve and coronary arteries, and establishing a stable and robust proximal anastomotic area. If an intimal tear or imprecise suture results in incomplete closure of the false lumen, continued blood flow can trigger root dilatation, which can lead to tearing of the sinusoidal dissection and uncontrollable bleeding. This situation can compress the coronary artery openings, leading to difficult cardiac arrest, and can even jeopardize the patient's life. Therefore, meticulous attention to every detail of the aortic root during surgery is essential to prevent postoperative complications and to ensure the success of the procedure [14].

The aortic wall is extremely fragile due to entrapment lesions, often requiring reinforcement to ensure a safe closure. In 1966, Gerbode et al. [15] introduced the “sandwich” method of aortic root reinforcement, utilizing Teflon felt strips on both the inner and outer sides of the diseased aortic wall to securely affix the detached membranes, thus forming a “sandwich”-like structure. This technique employs Teflon felt strips to securely join the separated inner and outer aortic membranes, forming a robust structure. It has undergone improvements since its introduction and remains widely used. For instance, Cacheraet al. [16] suggested using wider felt strips; in China, Prof. Xu Zhiyun applied this method to completely cover the entire noncoronary sinus and eliminate its pseudo-lumen, effectively preventing tears in the intercalated tissues and blood seepage. However, the expansion of the felt strips may complicate needle entry and suturing, posing risks of aortic stenosis, hemolysis, and thromboembolism [17–19]. These concerns must be carefully considered and addressed in clinical practice. Consequently, Gaeta et al. [20] began using polyester artificial vascular strips, which offer better flexibility and maneuverability, thus alleviating needle entry difficulties and facilitating smoother suturing. Moreover, polyester material exhibits greater stability in response to blood swelling, potentially reducing the risk of aortic complications. Fang et al. [26] compared the efficacy of a modified eversion anastomosis technique versus the conventional patch sandwich technique for proximal aortic anastomosis. Their results demonstrated that the novel eversion suture technique significantly reduced proximal anastomosis time (38 ± 12 min vs. 58 ± 20 min, *P* < 0.001), along with shorter cardiopulmonary bypass and aortic cross-clamp durations, and less intraoperative blood loss. In the study by Biancari et al. [27] on the application of the David procedure in type A aortic dissection, the aortic cross-clamp time was longer with the David procedure compared to our surgical approach (170 ± 65 min vs. 151.0 [112.0–209.0] min). Additionally, the David procedure was associated with a higher incidence of postoperative complications (87 vs. 25%), elevated perioperative mortality (11.3 vs. 7.1%), but a lower rate of postoperative hemodialysis (7.0 vs. 10.7%). Prof. Kexiang Liu employed artificial vascular sheets measuring 1.5–2.0 cm in width to reinforce the aortic root stump during surgery, demonstrating significant improvements in procedural outcomes [6]. A 4–0 thread continuous horizontal mattress, transmural foldback suture was utilized at the lower edge of the “sandwich” structure at the sinotubular junction and at the upper edge in the plane of the aortic dissection. Additionally, 3–5 longitudinal mattress sutures were employed within the sandwich structure to separate the false lumen. This method was employed in our previous clinical practice, proving to be easy to perform, master, and effective, thus providing reliable technical support for the operation. Aortic valve closure insufficiency, often resulting from acute type A aortic dissection, is primarily due to intimal avulsion at the leaflet junction, with the right atrioventricular junction avulsion being particularly common. In patients exhibiting normal aortic valve leaflet activity, absence of organic lesions, and a normal aortic valve structure without significant left ventricular enlargement on preoperative ultrasound, aortic valve junction suspension can typically be conducted intraoperatively using a 4–0 double-ended needle with spacers in a mattress suture technique to achieve satisfactory aortic valvuloplasty outcomes. Following the suspension, a water injection test is conducted to assess aortic valve closure and confirm recovery of valve function. Furthermore, employing continuous sutures on the upper and lower margins can induce a ring contraction effect on the sinotubular junction widening due to entrapment, positively impacting the restoration of sinus morphology and enhancing aortic valve function. The study results indicated that the procedure was effective. In this cohort, 23 patients presented with varying degrees of aortic valve closure insufficiency preoperatively; postoperatively, aortic regurgitation was completely resolved in 13 patients, while the remainder exhibited only mild aortic regurgitation. This suggests that aortic valve junction suspension possesses a high success rate and significant clinical value in managing aortic valve closure insufficiency attributable to entrapment.

One patient in our group succumbed postoperatively due to coronary causes. In this case, a significant dilatation of the sinus epithelium, but not of the intima, was observed intraoperatively, resulting in a mismatch between the inner and outer dimensions. The entrapment involved the right coronary artery opening, identified as a Neri A-type lesion; however, this opening was not reinforced intraoperatively. Despite smooth cardiac rebeating and shutdown during the procedure, a significant hematoma was discovered in the aortic sinus and part of the right sinus during chest closure. The patient's circulation remained stable during the postoperative night; however, the following morning, he developed anuria accompanied by unsustainable blood pressure and elevated central venous pressure. Bedside ultrasound revealed right ventricular distension and dyskinesia, and additional bedside electrocardiography along with myocardial injury marker testing confirmed an inferior wall myocardial infarction. We hypothesized that the cause was likely due to a sinus hematoma compressing the right coronary artery opening. Reflecting on this case, the patient's sinus dilated to a diameter exceeding 45 mm, whereas the intima-media was not significantly dilated, resulting in a severe mismatch that precluded effective use of the “sandwich” method for root angioplasty. Upon opening the aorta, the sinus membrane, lacking support from the outer membrane, is susceptible to the high-pressure blood flow from the aortic root, potentially leading to endothelial tearing at the needle site and blood ingress into the sandwich's pseudocavity, with serious consequences. For these patients, in the absence of primary valve pathology and without undergoing root replacement or David’s surgery, it is advisable to reinforce the inner lining of the uncinate sinus with a patch, or to customize shims to the shape of the uncinate sinus, lining both its inner and outer sides. The “sandwich” reinforcement of the uncrowned sinus effectively covers and eliminates its pseudocavity, thus diminishing the risk of postoperative complications and enhancing the procedure’s safety and efficacy [21–25].

There are some limitations. This study is a retrospective case series report, limited by the small number of cases and the lack of mid- to long-term follow-up data. Therefore, further research is required to evaluate the long-term efficacy of this technique, particularly regarding the incidence and progression of residual dissection in the aortic sinus. The modified “sandwich” technique using synthetic vascular patches for root reconstruction was performed above the level of the sinotubular junction. However, the separation between the intima and adventitia in the aortic sinus persists after dissection. Consequently, additional observation and investigation are necessary to assess the incidence of postoperative residual dissection in the sinus and the progression of sinus-related pathologies over the long term. Therefore, we will extend the follow-up period and establish a standardized surveillance protocol for patients undergoing the modified sandwich technique, including annual contrast-enhanced CT and echocardiography to monitor residual dissection in the aortic sinus. Furthermore, a multicenter randomized controlled trial will be conducted comparing different surgical approaches for acute type A aortic dissection (AADA) (modified sandwich technique vs. David procedure vs. conventional Bentall procedure). Primary endpoints will include 5-year major adverse cardiovascular event (MACE)-free survival and sinus-related complication rates. By expanding the sample size, this study aims to provide a more robust evaluation of the therapeutic efficacy of the novel technique.

In the surgical management of acute type A aortic dissection (AADA), the use of the modified “sandwich” technique with synthetic vascular patches for aortic root reconstruction has demonstrated favorable clinical outcomes. This technique is straightforward to implement and easy to master, significantly reducing the risk of bleeding and tearing at the aortic root anastomosis, thereby effectively preventing severe intraoperative and postoperative bleeding complications. The findings of this study indicate that the majority of patients experienced satisfactory postoperative recovery with a low incidence of complications, and only a small number of patients exhibited residual dissection or other complications. Overall, the modified “sandwich” technique has shown significant efficacy in the surgical treatment of acute type A aortic dissection and is worthy of further promotion and application in clinical practice.

## Conclusion

In conclusion, the application of the “modified sandwich” method in acute Stanford A aortic dissection root planning has been satisfactory. This method is straightforward to implement, simple to master, and highly valuable for widespread adoption, potentially enabling more hospitals to perform this surgery and thereby extending valuable treatment time for patients.

## Data Availability

All datasets were used and/or analyzed during the current study are available from the corresponding author on reasonable request.

## Abbreviations

AADA: Acute type A aortic dissection
CTA: Computed tomography angiography
EF: Ejection fraction
HTK: Histidine-tryptophan-ketoglutarate (cardioplegic solution)
ICU: Intensive care unit
Cr: Creatinine
BUN: Blood urea nitrogen
CT: Computed tomography
SPSS: Statistical package for the social sciences
MST: Modified sandwich technique
BMI: Body mass index

## References

[1] Saw LJ, Lim-Cooke MS, Woodward B et al (2020) The surgical management of acute type A aortic dissection: current options and future trends. J Card Surg 35:2286–2296. 10.1111/jocs.14733 32598525 10.1111/jocs.14733

[2] Gao F, Shi Z, He X et al (2022) The short-term efficacy of adventitial inversion with graft eversion anastomosis for the reconstruction of the aortic sinus in the root treatment of aortic dissection. Front Cardiovasc Med 9:845040. 10.3389/fcvm.2022.845040 36072881 10.3389/fcvm.2022.845040 PMC9441655

[3] Suzuki R, Kurazumi H, Nawata R et al (2022) Intimal-protected adventitial inversion technique accelerates the obliteration of a patent false lumen. J Card Surg 37:2600–2606. 10.1111/jocs.16720 35771215 10.1111/jocs.16720

[4] Koshiyama H, Okubo S, Futagami D et al (2018) Adventitial wrap technique for acute type A aortic dissection. Ann Thorac Surg 106:e329–e331. 10.1016/j.athoracsur.2018.05.076 29966593 10.1016/j.athoracsur.2018.05.076

[5] Kaya E (2018) Reinforcement of suture lines with aortic eversion in aortic replacement. Cardiovasc J Afr 29:12–45. 10.5830/CVJA-2017-008 29443351 10.5830/CVJA-2017-008 PMC6002793

[6] Zhu C, Piao H, Wang Y et al (2019) A new aortic root reinforcement technique for acute type A aortic dissection surgery. Int Heart J 60:1131–1136. 10.1536/ihj.18-609 31484859 10.1536/ihj.18-609

[7] Osada H, Minatoya K (2023) Overview of acute type A dissection in Japan. Indian J Thorac Cardiovasc Surg 39:280–286. 10.1007/s12055-023-01548-x 38093936 10.1007/s12055-023-01548-x PMC10713900

[8] Chen P, Chen M, Chen L et al (2022) Risk factors for severe acute kidney injury post complication after total arch replacement combined with frozen elephant trunk, in acute type A aortic dissection. Cardiovasc Diagn Ther 12:880–891. 10.21037/cdt-22-313 36605080 10.21037/cdt-22-313 PMC9808119

[9] Zhou H, Wang G, Yang L et al (2018) Acute kidney injury after total arch replacement combined with frozen elephant trunk implantation: incidence, risk factors, and outcome. J Cardiothorac Vasc Anesth 32:2210–2217. 10.1053/j.jvca.2018.02.026 29571643 10.1053/j.jvca.2018.02.026

[10] Rylski B, Schilling O, Czerny M (2023) Acute aortic dissection: evidence, uncertainties, and future therapies. Eur Heart J 44:813–821. 10.1093/eurheartj/ehac757 36540036 10.1093/eurheartj/ehac757

[11] Wang W, Duan W, Xue Y et al (2014) Clinical features of acute aortic dissection from the registry of aortic dissection in China. J Thorac Cardiovasc Surg 148:2995–3000. 10.1016/j.jtcvs.2014.07.068 25433882 10.1016/j.jtcvs.2014.07.068

[12] Zhao R, Qiu J, Dai L et al (2022) Current surgical management of acute type A aortic dissection in China: a multicenter registry study. JACC Asia 2:869–878. 10.1016/j.jacasi.2022.08.009 36713764 10.1016/j.jacasi.2022.08.009 PMC9876964

[13] Fang Z, Li H, Warburton TM et al (2022) Surgical repair of two kinds of type A aortic dissection after thoracic endovascular aortic repair. Front Cardiovasc Med 9:849307. 10.3389/fcvm.2022.849307 35433848 10.3389/fcvm.2022.849307 PMC9005800

[14] Yang B, Malik A, Waidley V et al (2018) Short-term outcomes of a simple and effective approach to aortic root and arch repair in acute type A aortic dissection. J Thorac Cardiovasc Surg 155:1360-1370.e1361. 10.1016/j.jtcvs.2017.11.089 29397965 10.1016/j.jtcvs.2017.11.089

[15] Gerbode F, Semb GS, Hill JD et al (1966) Aneurysms of the ascending aorta. A method of reconstructing the aortic root. Ann Thorac Surg 2:525–531. 10.1016/s0003-4975(10)66612-x 5934067 10.1016/s0003-4975(10)66612-x

[16] Cachera JP, Vouhe PR, Loisance DY et al (1981) Surgical management of acute dissections involving the ascending aorta. Early and late results in 38 patients. J Thorac Cardiovasc Surg 82:576–584 7278349

[17] Svensson LG, Crawford ES, Hess KR et al (1990) Dissection of the aorta and dissecting aortic aneurysms. Improving early and long-term surgical results. Circulation 82:IV24-38 2225411

[18] Sakaguchi M, Takano T (2016) Hemolytic anemia caused by aortic flap and inversion of felt strip after ascending aorta replacement. J Cardiothorac Surg 11:117. 10.1186/s13019-016-0520-1 27484121 10.1186/s13019-016-0520-1 PMC4969649

[19] Sogawa M, Moro H, Namura O et al (2001) Thrombus on the intraluminal felt strip. A possible cause of postoperative stroke. Jpn J Thorac Cardiovasc Surg 49:333–335. 10.1007/BF02913145 11431957 10.1007/BF02913145

[20] Gaeta R, Lentini S, Tancredi F et al (2009) Surgery for acute aortic dissection: an easy and cheap method to reinforce the anastomosis. J Card Surg 24:173–174. 10.1111/j.1540-8191.2008.00712.x 18793240 10.1111/j.1540-8191.2008.00712.x

[21] Rahmath MRK, Boudjemline Y, Kamal RY (2020) Aortic atresia with interrupted aortic arch and bilateral arterial ductus: a successful initial palliation. Cardiol Young 30:1732–1734. 10.1017/S1047951120002942 33198834 10.1017/S1047951120002942

[22] Seguchi R, Horikawa T, Kiuchi R et al (2020) Successful two-stage treatment for coarctation of the aorta-postductal type and aortic regurgitation with thoracic endovascular aortic repair and aortic valve replacement. Ann Vasc Dis 13:414–417. 10.3400/avd.cr.20-00040 33391560 10.3400/avd.cr.20-00040 PMC7758586

[23] Tsutsumi K, Yasuda T, Ishida O (2020) Adult untreated coarctation of the aorta developing acute type B dissection. Asian Cardiovasc Thorac Ann 28:120–122. 10.1177/0218492319891028 31744305 10.1177/0218492319891028

[24] Chen LW, Wu XJ, Li QZ et al (2012) A modified valve-sparing aortic root replacement technique for acute type A aortic dissection: the patch neointima technique. Eur J Cardiothorac Surg 42:731–733. 10.1093/ejcts/ezs371 22743079 10.1093/ejcts/ezs371

[25] Tang Y, Liao Z, Han L et al (2017) Long-term results of modified sandwich repair of aortic root in 151 patients with acute type A aortic dissection. Interact Cardiovasc Thorac Surg 25:109–113. 10.1093/icvts/ivw416 28398529 10.1093/icvts/ivw416

[26] Fang C, Gao S, Ren X et al (2022) Comparison of two techniques in proximal anastomosis in acute type A aortic dissection. Front Cardiovasc Med 9:1047939 36386353 10.3389/fcvm.2022.1047939 PMC9643206

[27] Peterss S (2024) The David versus the Bentall procedure for acute type A aortic dissection. J Cardiovasc Dev Disease 11:370. 10.3390/jcdd11110370 39590213 10.3390/jcdd11110370 PMC11594449

